# Economic Impact of Cystic Echinococcosis in Peru

**DOI:** 10.1371/journal.pntd.0001179

**Published:** 2011-05-24

**Authors:** Pedro L. Moro, Christine M. Budke, Peter M. Schantz, Julio Vasquez, Saul J. Santivañez, Jaime Villavicencio

**Affiliations:** 1 Division of Healthcare Quality Promotion, Immunization Safety Office, Centers for Disease Control and Prevention, Atlanta, Georgia, United States of America; 2 College of Veterinary Medicine and Biomedical Sciences, Texas A&M University, College Station, Texas, United States of America; 3 Rollins School of Public Health, Emory University, Atlanta, Georgia, United States of America; 4 Portneuf Medical Center and Idaho State University, Pocatello, Idaho, United States of America; 5 Instituto Peruano de Parasitologia Clinica y Experimental, Lima, Peru; 6 Servicio Nacional de Sanidad Agraria, Ministerio de Agricultura, Lima, Peru; Case Western Reserve University School of Medicine, United States of America

## Abstract

**Background:**

Cystic echinococcosis (CE) constitutes an important public health problem in Peru. However, no studies have attempted to estimate the monetary and non-monetary impact of CE in Peruvian society.

**Methods:**

We used official and published sources of epidemiological and economic information to estimate direct and indirect costs associated with livestock production losses and human disease in addition to surgical CE-associated disability adjusted life years (DALYs) lost.

**Findings:**

The total estimated cost of human CE in Peru was U.S.$2,420,348 (95% CI:1,118,384–4,812,722) per year. Total estimated livestock-associated costs due to CE ranged from U.S.$196,681 (95% CI:141,641–251,629) if only direct losses (i.e., cattle and sheep liver destruction) were taken into consideration to U.S.$3,846,754 (95% CI:2,676,181–4,911,383) if additional production losses (liver condemnation, decreased carcass weight, wool losses, decreased milk production) were accounted for. An estimated 1,139 (95% CI: 861–1,489) DALYs were also lost due to surgical cases of CE.

**Conclusions:**

This preliminary and conservative assessment of the socio-economic impact of CE on Peru, which is based largely on official sources of information, very likely underestimates the true extent of the problem. Nevertheless, these estimates illustrate the negative economic impact of CE in Peru.

## Author Summary

Cystic echinococcosis (CE), caused by infection with the larval stage of the cestode *Echinococcus granulosus,* constitutes an important public health problem in Peru. Despite its high prevalence in endemic communities no studies have attempted to estimate the economic impact of CE in Peruvian society. We used official and published sources of epidemiological and economic information to estimate direct and indirect costs associated with livestock production losses and human disease. We also used disability adjusted life years (DALYs) which is an overall measure of disease burden, expressed as number of years lost due to ill-health, disability or early death due to CE. We found that the total estimated cost of human CE in Peru was U.S.$2,420,348 per year. Total estimated livestock-associated costs due to CE ranged from U.S.$196,681 to U.S.$3,846,754. An estimated 1,139 DALYs were also lost due to surgical cases of CE which is comparable to DALY losses from Amebiasis or Malaria in Peru. This conservative assessment found significant economic losses caused by this CE in Peruvian society. The findings of this study are important as these data can serve to prioritize those areas that may need to be targeted in a control program.

## Introduction

Cystic echinococcosis (CE) is caused by infection with the larval stage of the cestode *Echinococcus granulosus*. CE has a cosmopolitan distribution and is an important public health problem in sheep-raising areas of South America [1]. CE has a serious impact on human health and livestock production in Peru as demonstrated by epidemiological studies in endemic villages of Peru that have shown human infection prevalences ranging from 5.5% to 9.1% (see Table 1) [2], [3], with the prevalence of CE in sheep and cattle as high as 77% and 68%, respectively [2], [4]. Despite these levels of infection, CE is not a notifiable disease in Peru. CE not only causes disability and occasional death in humans, but also results in monetary losses due to treatment costs, lost wages, and animal productivity losses. An economic analysis of the losses due to CE in Uruguay revealed an estimated minimum cost of U.S.$ 2.9 million per year in losses in livestock productivity together with the cost of hospital treatment of human cases [5]. Despite the high levels of CE in Peru, no study has explored the socioeconomic or financial impact of this disease in Peru. Studies to estimate the economic impact of CE are important as they can serve as tools to help prioritize those areas that may need to be targeted in a control program.

**Table 1:** Human incidence and prevalence of cystic echinococcosis in Peru.

	Parameter	Reference
Peru		
Surgical incidence	1–2†	2,26
	3–4†	Current study
Regionally (Departments)		
Lima		
Vichaycocha	9.3%‡	3
Junín		
Overall surgical incidence	11.7 †	12
Rural farming area (SAIS Tupac Amaru)	5.4%‡	2
Pasco	5.5%‡	28
Ica – Chincha	32†	23

**†:** Cases per 100,000 inhabitants

**‡:** Ultrasound and chest-X-ray survey

Disability-adjusted life years (DALYs) have been used to quantify disease burden for a wide range of afflictions globally, and it was the primary metric used in the Global Burden of Disease (GBD) Study [6],[7]. DALYs offer a standardized, non-monetary measure of morbidity and mortality that is comparable across various conditions and geographic regions. The DALY also serves as a useful tool for resource allocation and cost-effectiveness analyses. Calculation of DALYs involves summing the years of life lost due to premature mortality and the years of a healthy life lost due to the disability caused by the infection in a specific population, accounting for the degree of incapacity resulting from various conditions (disability weights) and the relative importance of a healthy life at different ages (age-weights). DALYs have previously been applied to assess the economic impact of CE on a regional and global scale [8], [9]. Given the absence of data on this subject, this study was designed to provide a preliminary estimate of the socio-economic impact of CE in Peru.

## Materials and Methods

### Human CE epidemiological parameters

Data on annual reported cases of CE, by age, gender, and by cyst location (Table 2), were obtained from official government and published sources [10–12; Ministry of Health of Peru, unpublished data]. Government agencies in Peru record the number of new CE cases seen in outpatient clinics in public healthcare facilities, but no information is currently collected by the government on the number of CE cases that undergo surgical treatment. In order to estimate the number of cases of CE that underwent surgery nationally, we applied the proportion of CE cases who underwent surgical treatment from a known endemic area (for which data is available) [12] to the number of CE cases seen in outpatient clinics nationally. Thus, it was assumed that the proportion of CE cases who undergo surgery in this endemic region would also apply to the entire country (Table 2).

**Table 2:** Epidemiological parameters used to estimate the monetary and non-monetary burden of human CE in Peru, 2007.

Category	Value	Distribution	Ref.
Population of Peru	26,152,265	Fixed	27
Annual surgical incidence of CE (cases per 100,000)	3–4	Uniform	12,22
New CE outpatients	2189	Fixed	22
Asymptomatic or non-healthcare seeking cases	0–0.015–0.02	Triangular	See text
Gross national income (US $ per capita)	3,450	Fixed	9
Mortality (% of surgical cases)	1.9%	Log Normal	11
Location of CE cysts:			11
Liver	44.9%	Fixed	
Lung	32.5%	Fixed	
Liver and lung	22.6%	Fixed	
Distribution of CE by age (in years):			22
0–9	7.6%	Fixed	
10–19	19.2%	Fixed	
20–39	34.9%	Fixed	
40–49	12.1%	Fixed	
50–59	9.5%	Fixed	
60+	16.7%	Fixed	
Distribution of CE by sex:			22
Male	43.3	Fixed	
Female	56.7	Fixed	

Community-based data are not available to determine the proportion of asymptomatic or non-healthcare seeking CE patients in the Peruvian population. Therefore, the ratio of cases detected via abdominal ultrasound screening in a community survey to surgical incidence cases determined for the Junin Department was used to estimate the number of asymptomatic cases in Peru [2]. In this study, the ultrasound-based CE prevalence was 5.7%, with a surgical incidence of 127 cases per 10^5^ person years, resulting in a ratio of 44.9. When this ratio was applied to the countrywide Peruvian data, with an estimated surgical incidence of 3.6 cases per 10^5^ person years, the prevalence of undiagnosed cases was estimated to be 0.162%. Due to the uncertainty associated with this estimate, a triangular distribution with a minimum of 0%, a maximum of 0.2%, and a most likely value of 0.163% was used. The proportion of cases with post-surgical mortality was obtained from the literature, which suggested that approximately 1.9% of surgical cases in Peru die post-operatively (11).

A reduction in productivity was modeled for all healthcare seeking CE cases (surgical and outpatient) and non-healthcare seeking cases. Due to scarcity of data on productivity reduction associated with CE, an estimated 2% reduction, with a uniform distribution of 0% to 4%, was utilized based on the only available data [13]. Fatal cases were assigned a 100% loss of productivity. We did not estimate costs associated with non-surgical treatment of CE (other than PAIR which is not available in Peru) as we are not aware of any local or international publication that has assessed this form of treatment.

### Economic evaluation of human CE-associated losses

Mean overall costs per human surgical CE case, by cyst location (e.g., hepatic, pulmonary, or both), were obtained from published sources [14]. The cost of a surgical hepatic CE case was estimated at U.S.$645, the cost of a surgical pulmonary CE case was estimated at U.S.$831, and the cost of a surgical case of hepatic and pulmonary CE was estimated at U.S.$ 1,476. Cost per surgical patient included costs associated with the surgical procedure itself along with the cost of diagnostic testing, medications, and hospitalization. Per capita GNI (U.S.$3,450) for 2007 was used as the economic contribution of each affected patient and was, therefore, used to estimate productivity losses [15].

### Livestock CE epidemiological parameters

The prevalence of CE in livestock was calculated from official government abattoir data (Table 3) [10]. Reduction in livestock productivity, due to CE, has been documented by others and was assumed to occur in Peru [16]. Production-based losses attributable to infected cattle, sheep, goats, pigs, alpacas and llamas were, therefore, estimated based on previous reports [5] (Table 4).

**Table 3:** Prevalence of CE at slaughter.

Livestock species	Prevalence of cysts at abattoir inspection (national level)*	Distribution
Sheep	10%	Fixed
Goats	5.5%	Fixed
Cattle	6.0%	Fixed
Alpaca	2.0%–9.0%	Uniform
Llama	2.0%–9.0%	Uniform
Swine	3.8%	Fixed

*Ref 10

**Table 4:** Parameters used to estimate monetary losses due to CE in livestock, Peru 2007.

Livestock product	Current market value*	Annual production (kgs)	Production losses (%) b	Distribution
Liver:				
Cattle	$2.40/liver	___	100	Fixed
Sheep^a^	$0–$1.94/liver	___	100	Fixed
Meat				
Cattle	$2.21/kg	163,235,000	2.5–10	Uniform
Sheep	$2.50/kg	33,979,000	2.5–10	Uniform
Goat	$2.50/kg	6,721,000	2.5–10	Uniform
Pig	$2.22/kg	114,740,000	2.5–10	Uniform
Alpaca	$2.50/kg	9,400,000	2.5–10	Uniform
Llama	$2.50/kg	4,056,000	2.5–10	Uniform
Wool*				
Sheep	$12,420,444	10,885,000	10–20	Uniform
Alpaca	$11,224,825	3,881,000	10–20	Uniform
Llama	$1,030,423	724,000	10–20	Uniform
Cows' milk	$0.28/kg	1,575,277,00	0–5	Uniform

*Current market value in terms of total annual production in Peru, ref 17

a Ref 18

b Ref 16

### Economic evaluation of livestock CE-associated losses

Current market value for livers, meat, wool, and milk were obtained from official sources (Table 4) [17]. However, the price of a sheep liver was not available. Therefore, the maximum cost of a sheep liver was assumed to be proportional to the cost of cattle liver based on the proportion found in a previous study conducted in Tunisia where cattle liver was found to be 1.8 times more valuable than sheep liver [18]. Due to uncertainty, a uniform distribution with a minimum of no economic losses per infected sheep liver to a maximum of U.S.$1.94 per infected sheep liver was modeled. Losses from liver condemnation, defined as the action of preventing the sale of livers deemed unfit for human consumption (cattle and sheep), meat production losses (all species), decrease in wool value (sheep, alpacas, llamas), and decreased milk production (cattle) were calculated using production losses caused by CE as previously described [5], [16] (Table 4).

### Application of DALYs to human surgical CE cases

The DALY formula was applied to age and gender stratified human CE surgical incidence data. The DALY is made up of two parts; years of life lost due to mortality (YLL) and years of life lost due to time lived in a disability state (YLD). The formulas for YLL and YLD are:
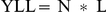
where N  =  number of deaths per age-sex group, L  =  remaining life expectancy at age of death
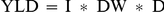
where I  =  age and sex specific estimates of incidence, DW  =  disability weight, D  =  average duration of disability.

Disability weight for CE was assigned a multinomial distribution based on numerous retrospective studies evaluating postoperative outcome. In accordance with previous studies, the percentage of patients projected to improve after surgery was assigned a disability weight of 0.200 for 1 year, the percentage of patients projected to have substantial postsurgical complications was assigned a disability of 0.239 for 5 years, the percentage of patients projected to have recurrent disease was assigned a disability of 0.809 for 5 years, and the percentage of patients projected to die postoperatively were assigned a disability of 1 (indicating death) for the remainder of their predicted lifespan [7]. Since malignant neoplasms of the respiratory tract (the best surrogate for CE of the lungs) have a similar disability weight at diagnosis (0.150) to that used previously for hepatic CE (0.200) and the same disability weights for sequelae, CE cases were assigned the same spectrum of disability weights regardless of lesion site. This also allows for estimates from this study to be comparable to previously published estimates for CE burden.

### Analysis

Spreadsheet models were constructed in Excel (Microsoft, Redmond, WA) utilizing the @Risk (Palisade Corp., Ithaca, NY) statistical add-in. Values were selected across the various assigned distributions and summed using Latin Hypercube sampling techniques. A total of 10,000 simulations were performed and the mean and 95% confidence intervals (CI) obtained for annual losses. Step-wise linear regression of the estimated CE-associated costs against the input parameters was conducted to assess the impact of individual parameters on the overall cost estimate.

This study was exempted from human subjects review by the Institutional Review Board of the Centers for Disease Control and Prevention because no personal identifiers were collected.

## Results

### Human health costs

Human health costs resulting from CE can be observed in table 5. Direct costs associated with surgical treatment of CE were estimated to account for U.S.$ 836,064 (95% CI:731,271–948,236) per year. Human health costs resulting from lost productivity were estimated at U.S.$1,592,764 (95% CI:310,664–3,947,315) annually. The total estimated cost of human surgical CE cases and productivity losses from non-surgically treated cases in Peru was estimated at U.S.$ 2,420,348 (95% CI:1,118,384–4,812,722) per year.

**Table 5:** Costs associated with human cystic echinococcosis in Peru.

Category of loss	Median(in U.S.$)	95% CI(in U.S.$)
Surgical cases of hepatic CE	191,223	167,254–216,878
Surgical cases of pulmonary CE	340,487	297,810–386,169
Surgical cases of hepatic/pulmonary CE	304,354	266,206–345,188
Mortality-associated productivity losses	45,343	37,187–55,875
Productivity losses (surgical cases)	47,563	4,357–91,422
Productivity losses (outpatients)	115,704	12,031–218,097
Productivity losses (asymptomatic cases)	1,370,468	121,685–3,747,867
Total losses	2,420,348	1,118,384–4,812,722

### Animal health costs

The estimated losses resulting from liver condemnation and production losses in each animal species are detailed in table 6. Total estimated losses due to CE ranged from U.S.$196,681 (95% CI:141,641–251,629) if only direct losses (i.e., cattle and sheep liver losses) are taken into consideration to U.S.$3,846,754 (95% CI:2,676,181–4,911,383) if all production losses evaluated (liver losses, decreased carcass weight, wool losses, decreased milk production) are accounted for.

**Table 6:** Livestock-associated economic losses due to cystic echinococcosis in Peru.

Category of loss	Median (in U.S.$)	95% CI (in U.S.$)
Direct losses (liver losses)	196,681	141,641–251,629
Meat production losses	2,675,475	1,777,403–3,532,182
Wool production losses	287,541	203,345–380,944
Milk production losses	655,472	62,955–1,267,465
Total losses	3,846,754	2,676,181–4,911,383

### Disability adjusted life years (DALYs)

The estimated number of DALYs lost due to surgical CE in Peru was estimated at 1,139 (95% CI: 861–1,489). Table 7 shows DALYs lost for surgical cases of CE in comparison with other infectious diseases in Peru as well as estimated DALYs lost due to CE in other South American countries (Argentina, Brazil, Chile and Uruguay).

**Table 7:** Estimated DALY's associated with surgical cases of cystic echinococcosis and selected infectious diseases^a^ in Peru.

Condition	Total	Males	Females
HIV/AIDS	20,741	16,062	4,679
Hepatitis B	4,398	2,734	1,664
Bartonellosis	2,540	1,944	596
Leishmaniasis	1,787	1,441	346
Amebiasis	1,281	705	576
Malaria	1,239	667	572
**Cystic echinococcosis (South America)** b	**1,766**	**NA**	**NA**
**Cystic echinococcosis**	**1,139**	**491**	**648**
Pertussis	1,106	605	501
Yellow Fever	703	631	72

a Ref 24

b DALYS for surgical cases of CE in Argentina, Brazil, Chile and Uruguay (Ref 19)

NA, not available

### Sensitivity analysis

Sensitivity analysis for monetary losses associated with CE, indicated that human productivity losses (0.691), the estimated number of sub-clinical cases (0.447), losses due to decreased cattle carcass weight (0.339), and losses due to decreased milk production in cattle (0.281) had the largest coefficient values (in brackets) and, therefore, the greatest impact on the overall estimate on monetary losses due to CE in Peru.

## Discussion

This is the first study to provide an assessment of the economic effects of CE in humans and livestock in Peru. Studies that estimate the economic burden of CE can be very important as they identify specific areas that may need to be addressed by policy makers responsible for the design and implementation of CE control programs. The economic impact of CE has been assessed in South American countries where CE is endemic [5], [19]. A recent study in Argentina, Brazil, Chile and Uruguay demonstrated total adjusted human and animal losses of US $ 108,276,378-$ 146,580,935 for all four countries combined [19]. However, it is difficult to make direct comparisons with other countries due to demographic and epidemiological differences. In addition, there is no standard approach recognized when estimating economic costs for CE in human and animal populations. For example, treatment costs for non-surgical CE patients were not estimated and included in the present study. Per capita GNI was also used in this study to account for human productivity losses rather than taking into account income losses for those who work outside the home versus those who are not officially employed. In addition, losses due to decreased fecundity were not included due to the lack of information on the true impact of CE on reproduction. In addition, livestock were not separated by age since CE prevalence data is not available for different age groups of domestic livestock. Finally, farmer investment was also not taken into consideration. In the present study, total estimated losses due to CE were U.S. $ 6,357,027 (95% CI: 4,422,699–8,788,182) of which two-thirds comprised human economic losses and the other third were animal production losses. This is a very conservative estimate based to a large extent on official sources of information which very likely underestimate the true extent of the problem. Nevertheless these estimates illustrate the negative economic impact of CE in Peru.

In Peru, surgery remains the treatment of choice for CE. Surgical treatment of CE is a costlier alternative than the less invasive puncture-aspiration-injection-reaspiration (PAIR) technique or benzimidazole use. In Argentina, a country where CE is endemic, the average cost of conventional surgery for CE of the liver was $5,936 in comparison to the cost of PAIR at $1,988 and $1,350 for albendazole treatment [20]. PAIR is not available in Peru and use of benzimidazoles is reserved for prophylactic use prior to surgery [21]. Use of less costlier procedures in Peru such as PAIR and sole chemotherapeutic treatment could result in lower overall treatment costs. Our estimate of the cost of surgical treatment for CE is based on the cost incurred in a public hospital located in Lima city. Costs may vary in other areas of Peru, especially in endemic areas. Therefore, our estimates may not reflect the true cost of surgical treatment in the country. In addition, this estimate does not take into account an important number of patients who receive treatment at private clinics where the cost of surgical treatment can be 4 or more times higher than in public hospitals. In one study in an endemic area of Peru, 8% of patients received treatment in private clinics compared to 44% in public hospitals and 47% in hospitals of the national health care system [12]. Our estimates of treatment costs for CE underestimates the true cost of human CE as we did not include the cost of benzimidazoles given the absence of data on sole benzimidazole use against CE in Peru.

An important limitation of this study is the absence of current data on the incidence or prevalence of CE in humans and animals. Available sources of data have not been updated for the last 20–30 years. Although the Peruvian Ministry of Health has regularly collected information on outpatient consultations due to CE since 2000, it is not known how many of these cases were confirmed and the type of treatment received. Data on CE condemned livestock viscera at the national level is collected by the Peruvian Ministry of Agriculture from inspections performed in abattoirs. However, a substantial number of livestock are home slaughtered, particularly in areas endemic for CE. In addition, younger animals, which are less likely to be infected, tend to be slaughtered in abattoirs. Therefore, these official sources of information may greatly underestimate the real prevalence of infection. For example, based on official publications, the incidence of human CE in the endemic Department of Junin was 24 cases per 100,000 during 2002 [22]. However, epidemiological studies conducted in Junin have demonstrated that the surgical incidence can reach 127 cases per 100,000 [2]. Similarly, official records showed that in the coastal city of Chincha the incidence of human infection was 10 cases per 100,000 during 2005 [22], but an epidemiological investigation revealed a surgical incidence of 32 cases per 100,000 population [23]. For livestock, official sources indicated a prevalence of sheep CE of 10% for 2002 in the Department of Junin [10]. However, epidemiological studies in abattoirs in this same area showed that as many as 77% of sheep were infected [4].

We estimated that 1,139 (95% CI: 861–1,489) DALYs are lost annually in Peru due to surgical cases of CE, which is comparable to CE-associated DALYs lost for Argentina, Brazil, Chile and Uruguay combined [19]. DALYS lost due to CE were also found to be similar to other conditions such as malaria, amoebiasis, and leishmaniasis [24]. These infectious diseases are better monitored than CE and thus their DALYs estimates are likely to better capture the true impact of those diseases on the country. In the case of CE, the absence of updated information on the frequency of human CE hampers the calculation of this socio-economic indicator. However, the number of DALYs estimated to be lost in this study is very conservative since only surgical cases of CE were evaluated.

The findings of the present economic evaluation, although conservative, highlight the monetary losses caused by CE in the Peru. Control programs could potentially prevent many of these losses in a cost-effective manner. For example, a control program implemented in the highly endemic region of La Rioja, Spain based on a combination of owned dog deworming, stray dog control, and safe ruminant carcass disposal resulted in a positive cost/benefit ratio after 8 years. This ratio increased to 1.96 by the conclusion of the 14-year program [25]. Control programs have been previously implemented in Peru. For example, a pilot control program was conducted in Peru during the 1970′s which was later abandoned due largely to political instability. However, the program succeeded in reducing the number of infected dogs from 36% in 1975 to 1.6% in 1980 and the number of infected sheep from 65% in 1976 to 37% in 1980 [2], [26]. There is urgent need to implement better surveillance systems to monitor the incidence/prevalence of echinococcosis in humans and animals in Peru so that they may be used as part of new control efforts for CE. In summary, CE is an important but neglected zoonotic disease in Peru which significantly affects the economy of the country.

## Supporting Information

Alternative Language Abstract S1 Spanish translation of the abstract by PLM.
(DOCX)
