# Supplementary material for: Economic Impact of Cystic Echinococcosis in Peru
Source: PLoS Negl Trop Dis. 2011 May 24;5(5):e1179. doi: 10.1371/journal.pntd.0001179 (PMC3101191; doi:10.1371/journal.pntd.0001179)
Supplement: Alternative Language Abstract S1 — Spanish translation of the abstract by PLM. (DOCX) [file pntd.0001179.s001.docx]

**Impacto Económico de la Equinococcosis Quística en el Perú**

**Antecedentes:** la equinococosis quística (EQ) constituye un importante problema de salud pública en el Perú. Sin embargo, ningún estudio ha tratado de estimar el impacto monetario y no monetario de la EQ en la sociedad peruana. **Métodos:** se utilizaron fuentes oficiales y publicadas de información epidemiológica y económica para estimar los costos directos e indirectos asociados con las pérdidas de la producción ganadera y la enfermedad humana, además de los años de vida ajustados por discapacidad (AVAD) perdidos asociados a la cirugía por EQ. **Resultados:** el costo total estimado de la EQ humana en el Perú fue de EE.UU. $ 2, 420,348 dólares (95% IC: 1, 118,384 - 4, 812,722) por año. Los costos totales estimados asociados a la ganadería debido a la EQ varió de EE.UU. $ 196,681 dólares (95% IC :141,641 - 251, 629), cuando sólo las pérdidas directas (destrucción de hígado de ganado vacuno y ovino) se tuvieron en cuenta a EE.UU. $ 3,846,754 dólares (IC del 95% :2,676,181 - 4, 911,383 ) si las pérdidas adicionales de producción (la condena del hígado, disminución de peso de carcasa, pérdidas de lana, disminución de la producción de leche) se tuvieron en cuenta. Se estima que 1,139 (95% IC: 861 -1,489) AVAD también se perdieron debido a los casos quirúrgicos de EQ. **Conclusiones:** esta evaluación preliminar y conservadora del impacto socio-económico de la EQ en el Perú, que se basa en gran medida en fuentes oficiales de información, muy probablemente subestima la verdadera magnitud del problema. Sin embargo, estas estimaciones ilustran el impacto económico negativo de EQ en el Perú.
